# Inflammatory Conditions Dictate the Effect of Mesenchymal Stem or Stromal Cells on B Cell Function

**DOI:** 10.3389/fimmu.2017.01042

**Published:** 2017-08-28

**Authors:** Franka Luk, Laura Carreras-Planella, Sander S. Korevaar, Samantha F. H. de Witte, Francesc E. Borràs, Michiel G. H. Betjes, Carla C. Baan, Martin J. Hoogduijn, Marcella Franquesa

**Affiliations:** ^1^Nephrology and Transplantation, Department of Internal Medicine, Erasmus MC, University Medical Center, Rotterdam, Netherlands; ^2^REMAR Group and Nephrology Service, Germans Trias i Pujol Health Science Institute & University Hospital, Badalona, Spain; ^3^Department of Cell Biology, Physiology and Immunology, Universitat Autònoma de Barcelona, Barcelona, Spain

**Keywords:** B cell, immunomodulation, mesenchymal stem cell, plasmablast, regulatory B cell, indoleamine 2,3-dioxygenase

## Abstract

The immunomodulatory capacity of mesenchymal stem or stromal cells (MSC) makes them a promising tool for treatment of immune disease and organ transplantation. The effects of MSC on B cells are characterized by an abrogation of plasmablast formation and induction of regulatory B cells (Bregs). It is, however, unknown how MSC interact with B cells under inflammatory conditions. In this study, adipose tissue-derived MSC were pretreated with 50 ng/ml IFN-γ for 96 h (MSC–IFN-γ) to simulate inflammatory conditions. Mature B cells were obtained from spleens by CD43^−^ selection. B cells were co-cultured with MSC and stimulated with anti-IgM, anti-CD40, and IL-2; and after 7 days, B cell proliferation, phenotype, Immunoglobulin-G (IgG), and IL-10 production were analyzed. MSC did not inhibit B cell proliferation but increased the percentage of CD38^high^ CD24^high^ B cells (Bregs) and IL-10 production, while MSC–IFN-γ significantly reduced B cell proliferation and inhibited IgG production by B cells in a more potent fashion but did not induce Bregs or IL-10 production. Both MSC and MSC–IFN-γ required proximity to target cells and being metabolically active to exert their effects. Indoleamine 2,3 dioxygenase expression was highly induced in MSC–IFN-γ and was responsible of the anti-proliferative and Breg reduction since addition of tryptophan (TRP) restored MSC properties. Immunological conditions dictate the effect of MSC on B cell function. Under immunological quiescent conditions, MSC stimulate Breg induction; whereas, under inflammatory conditions, MSC inhibit B cell proliferation and maturation through depletion of TRP. This knowledge is useful for customizing MSC therapy for specific purposes by appropriate pretreatment of MSC.

## Introduction

B cells contribute to immunological diseases in various ways by production of auto-antibodies, presentation of auto-antigen, and secretion of inflammatory cytokines. In the context of post solid organ transplantation, B cells mediate humoral rejection by the production of donor-specific human leukocyte antigen (HLA) antibodies (DSAs) and provide co-stimulatory signals to T cells (1, 2). On the other hand, a population of regulatory B cells (Bregs) has been described that can regulate immune responses mainly via the secretion of IL-10 (3, 4). Bregs have been shown to be involved in suppressing autoimmune reactions as well as in maintaining transplant tolerance (5, 6). Current treatments for B cell-mediated disease are mainly based on global B cell depletion, thereby eliminating pathogenic B cells as well as Breg subsets. A more refined modulation of B cell activity could prove beneficial for patient treatment.

Mesenchymal stem or stromal cells have potent immunomodulatory properties and target the proliferation and differentiation of a variety of immune cells (7). The effect of MSC on T cells has been extensively studied but also regulation of natural killer cells (8), macrophages (9), dendritic cells (10), and more recently B cells by MSC has drawn attention. Previously, we have shown that MSC can abrogate plasmablast formation and induce IL-10^+^ and CD19^+^ CD38^high^ CD24^high^ B cells (11), which are the two main signatures to define Bregs (12). However, it appears that the nature of the immunosuppressive and anti-proliferative effects of MSC on lymphocytes is dependent on the inflammatory microenvironment (13–16). In particular, IFN-γ has a prominent role in potentiating the anti-proliferative capacity of MSC via the induction of indoleamine 2,3-dioxygenase (IDO) activity (17) and contact dependent mechanisms of action (18, 19). Priming of MSC with inflammatory factors is likely to occur *in vivo* as MSC-treated patients often suffer from acute or chronic inflammatory diseases. MSC infused in patients might encounter an inflammatory environment that could influence the immunomodulatory effect of MSC.

We previously showed that B cell proliferation is increased when B cells are stimulated by an anti-CD40 + anti-IgM + IL-2 cocktail as well as with activated T cells. MSC reduced B cell proliferation induced by stimulated T cells but not by the cocktail in the absence of T cells (11). In our previous work, we hypothesized that the anti-proliferative effect of MSC on B cells in the presence of activated T cells was due to the secretion of IFN-γ by activated T cells and the subsequent activation of MSC. In this study, we examined how IFN-γ affected the immunomodulatory role of MSC on B cells by comparing the effects of MSC and IFN-γ treated MSC on B cell proliferation and differentiation into plasmablasts or IL-10 producing Bregs.

## Materials and Methods

### Isolation and Culture of Human Subcutaneous Adipose Tissue MSC

Subcutaneous adipose tissue from healthy human donors that became available as a waste product during kidney donation procedures was collected after obtaining written informed consent as approved by the Medical Ethical Committee of the Erasmus University Medical Centre Rotterdam (protocol no. MEC-2006-190). The tissue was collected in minimum essential medium-α (MEM-α) (Sigma Aldrich, St. Louis, MO, USA) supplemented with penicillin (100 IU/ml), streptomycin (100 mg/ml) (1% P/S; Lonza, Verviers, Belgium), and 2 mM L-glutamine (Lonza) and stored at 4°C for 3–16 h. MSC were isolated as described previously (20). Briefly, adipose tissue was mechanically disrupted and digested enzymatically with 0.5 mg/mL collagenase type IV (Life Technologies, Paisley, UK) in RPMI 1640 Medium with glutaMAX (Life Technologies) for 30 min at 37°C under continuous shaking. Cultures were kept at 37°C, 5% CO_2_, and 95% humidity and refreshed weekly with MEM-α with 1% P/S, and 15% heat-inactivated fetal bovine serum (FBS; Lonza). At 90% confluence, adherent cells were removed from culture flasks by incubation in 0.05% trypsin-EDTA (Life Technologies, Bleiswijk, The Netherlands) at 37°C and cells used for experiments or frozen at −150°C until further use. MSC were used for experiments between passages 2 and 5 and their phenotypic markers and osteogenic and adipogenic potential were tested as described before (21). MSC from 19 different donors were used in the experiments.

### Stimulation of MSC

Mesenchymal stem or stromal cells were pretreated for 4 days with IFN-γ (50 ng/ml; Life technologies). For co-culture experiments, MSC were washed with phosphate buffered saline (PBS) and detached by incubation with 0.05% trypsin-EDTA before seeding them in 96 well-plates in Iscove’s Modified Dulbecco’s Medium (IMDM, Lonza) with 10% heat inactivated FBS. Phenotypical characteristics of MSC before and after IFN-γ were assessed measuring several markers on their surface: CD13-PeCy7 (clone L138), CD31-V450 (clone WM59), CD45-APC-H7 (clone 2D1), HLA-ABC-APC (clone G46-2.6), HLA-DR PerCP (clone L243) and CD73-PE (clone AD2; all BD Biosciences), CD90-APC (clone Thy-1A1), and CD105-FITC (clone 166707; all R&D Systems, Minneapolis, MN, USA) and PD-L1 PE (clone B7-H1; Biolegend, San Diego, CA, USA) by Flow Cytometry and optical microscopy morphology (Figure S1 in Supplementary Material).

### IDO Activity Measurement

The activity of IDO was determined by the measurement of L-kynurenine in the supernatant of four MSC cultures as described previously (22). Briefly, MSC were seeded at a density of 100,000 cells/well in a 6 wells plate and cultured for 4 days with or without 50 ng/mL IFN-γ. 30% trichloroacetic acid was added to the supernatant in a 1:3 ratio. Samples were incubated for 30 min at 50°C and spun down at 12,000 rpm for 5 min. Samples were plated in a 96 wells flat bottom plate and diluted 1:1 in Ehrlich reagent [200 mg 4-dimethylaminobenzaldehyde (Sigma-Aldrich, St. Louis, MO, USA) in 10 ml of glacial acetic acid]. Absorbance was read at 490 nm using a Wallac Victor2 1420 multilabel plate reader (Perkin Elmer, Waltham, MA, USA).

### Isolation of B Cells from Spleens

Spleens were obtained from post-mortal kidney donors (Erasmus MC Hospital, Rotterdam) and anonymously used for research purposes as described in article 13 of The Netherlands law of organ donation (*Wet op Orgaandonatie, WOD*). All samples and data were analyzed anonymously. Spleens were mechanically disrupted and filtered through a 70-µm cell strainer (Greiner Bio-one, Alphen a/d Rijn, The Netherlands) to obtain a single-cell suspension. Mononuclear cells, isolated using Ficoll-Paque (Amersham Pharmacia Biotech, Uppsala, Sweden) density gradient, were stored at −150°C until use. Upon thawing, quiescent B cells were isolated by negative selection using anti-CD43-magnetic beads (Miltenyi Biotec GmbH, Bergisch Gladbach, Germany) (23). Purity was determined by flow cytometry (FACS Canto II). Typically, cell suspensions consisted of >98% pure CD19^+^ B cells. B cells from spleens from 12 different donors were used it the experiments.

### B Cell Stimulation

B cells were co-cultured in IMDM-10%FBS with a cocktail to mimic antigen and T cell help: 10 mg/ml F(ab)2 anti-IgM (Jackson, ImmunoResearch laboratories, Inc., West Grove, PA, USA), 10^3^ IU IL-2 (Proleukin, Prometheus laboratories Inc., San Diego, CA, USA), and 5 mg/ml anti-CD40 agonistic monoclonal antibody (Bioceros, Utrecht, The Netherlands). In some of the experiments, 200 µM tryptophan (TRP, l-tryptophan, Sigma-Aldrich) was added to the stimulation cocktail to counteract the activity of IDO.

### Transwell (TW) Cultures

24-wells plates with 0.4 μm pore polycarbonate membrane inserts (Costar, Corning, Kennebunk, ME, USA) were used for the TW cultures. MSC were seeded on the membrane of the inserts and B cells were added to the lower chamber at a ratio MSC:B cells 1:5. After 7 days, inserts were removed; and B cells from the lower chamber were collected for further analysis and B cell subsets characterization.

### Heat Inactivated MSC

To study the effect of cell surface molecules but not the secreted factors, MSC were inactivated as previously described (24). Shortly, MSC were heated in suspension in PBS in parafilm-sealed tubes by 30 min incubation at 50°C in a temperature-regulated water bath. The inactivated cells were then washed and counted and used for further experiments.

### B Cell Subset Characterization

B cells were labeled by incubation with 5,6-carboxy-succinimidyl-fluoresceine-ester (CFSE) (Molecular Probes Invitrogen, Karlsruhe, Germany) for 10 min at 37°C.

After 7 days, B cells were collected and processed for flow cytometric analysis (FACS Canto II, Diva Software, BD Biosciences, San Jose, CA, USA), and supernatants were stored at −80°C for cytokine and Immunoglobulin-G (IgG) determination. The antibodies used for flow cytometry phenotyping were as follows: CD27-PE-Cy7 (clone 0323), CD38-PE (clone HB7), CD19-BV512 (clone HIB19) and CD24-APC (clone SN3 A5-2H1D) (eBioscience, San Diego, CA, USA), IL-10-Bv421 (Clone Jes3-9D7, Biolegend), and Via Probe for determination of cell viability (BD Biosciences, San Jose, CA, USA). After 7 days, proliferation of B cells was assessed by measuring CFSE dye dilution on a FACSCanto II flow cytometer (BD Biosciences). 12 h before harvesting the cells, Monensin (Golgi Stop, BD Biosciences) was added to the wells and the intracellular staining was performed without restimulation using Intrastain kit (Dako, Denmark).

### Measurement of Cytokine Secretion

Supernatants from MSC-B cell co-cultures kept at −80°C were thawed and used for measurement of cytokine levels. IL-10 was quantified using a Milliplex kit (Merck Millipore, Amsterdam, The Netherlands) according to manufacturer’s instructions. Human cytokine standards were provided by the kit and a standard curve was prepared from 10,000 to 3.2 pg/ml. Samples and standards mixed with antibody-coated magnetic beads were incubated overnight in a 96-well plate at 4°C under continuous agitation. Plates were washed and incubated with detection antibodies for 1 h. Finally, plates were washed and incubated with streptavidin-phycoerythrin for 30 min. The samples were measured on a Luminex 100/200 cytometer (Luminex, Austin, TX, USA) using Xponent software.

### IgG ELISA

Plates were coated with goat anti human Ig-UNLB (Southern Biotechnology Associates; Birmingham, AL, USA). Plates were washed with PBS 0.05% Tween and blocked with PBS 5% FBS for 2 h. Diluted samples and standard IgG (Sigma-Aldrich) were added to the plate and incubated for 90 min. IgG-HRP (My Biosource; San Diego, CA, USA) was used as a conjugate and 3,3,5,5-tetramethylbenzidine (TMB) was used to visualize bound IgG. Absorbance was read at 595 nm using a Wallac Victor2 1420 multilabel plate reader (Perkin Elmer, Waltham, MA, USA).

### RNA Expression Quantification

After 7 days of co-culture, B cells were recovered, pelleted in PBS–DEPC and snap frozen. RNA was isolated and 500 ng was used for cDNA synthesis as described previously (25). Gene expression was determined by real-time RT-PCR using universal PCR master mix (Life Technologies) and an assay-on-demand for IL-10 (Hs00174086.m1) (Applied Biosystems, Foster City, CA, USA) and analyzed on an ABI PRISM 7700 sequence detector (Applied Biosystems). Data are expressed as relative copy number of the PCR products with respect to the housekeeping gene GAPDH.

### Statistical Analysis

Data are expressed as means ± SEM. Significant differences within groups were calculated using repeated measures non-parametric analysis of variance (ANOVA: Friedman test) with Dunnett’s posttest performed by GraphPad Prism 5 software (GraphPad Software, San Diego, CA, USA). *P* values were indicated as * for *P* < 0.05; ** for *P* < 0.01; and *** for *P* < 0.001.

## Results

### IFN-γ-Pretreated MSC Inhibit B Cell Proliferation

Previously, we showed that the inhibition of B cell proliferation by MSC was dependent on the presence of T cells (11). We hypothesized that MSC needed to be activated by IFN-γ secreted by T cells to mediate their anti-proliferative effects on B cells.

**Figure 1:**
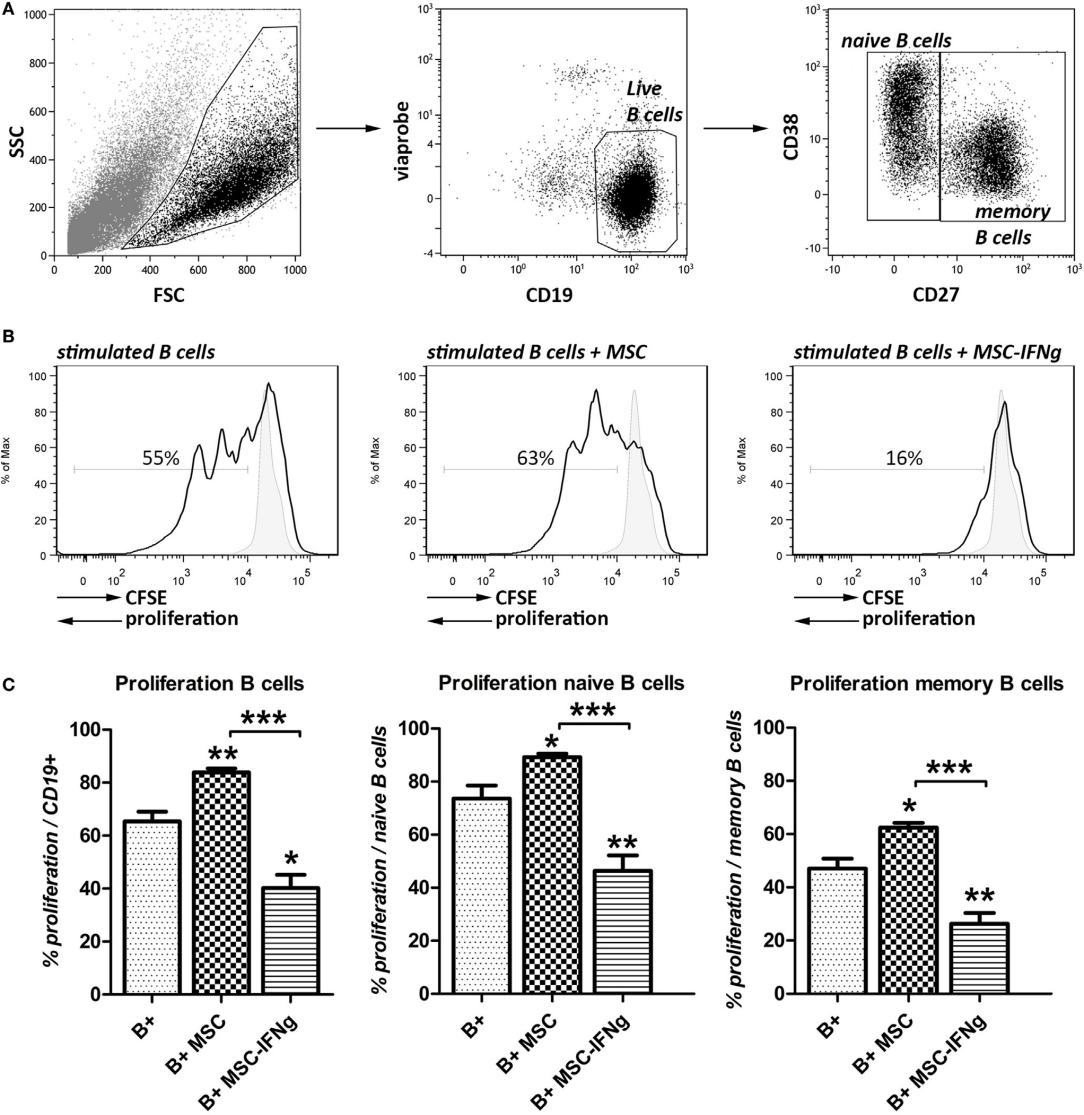
IFN-γ stimulated mesenchymal stem or stromal cells (MSC) reduce B cell proliferation. B cells from human splenocytes were stimulated for 7 days with anti-CD40, anti-IgM and IL-2 in the presence of adipose tissue-derived MSC or IFN-γ-pretreated MSC (MSC–IFN-γ). **(A)** Representative FACS plots of the gating strategy of live, naïve, and memory B cells based on intensity of CD27 expression. **(B)** Proliferation of B cells in the presence or absence of MSC or MSC–IFN-γ at a 5:1 (B cell:MSC) ratio was assessed through measurement of 5,6-carboxy-succinimidyl-fluoresceine-ester (CFSE) label dilution. Representative histograms were shown. Gray, solid histograms represent unstimulated B cells. **(C)** Percentage of proliferation of B cells (left graph), naïve B cells (middle graph), and memory B cells (right graph). Bars indicate mean ± SEM of three experiments with three different MSC cultures and three different B cell donors.

Here, we analyzed the anti-proliferative capacity of MSC and IFN-γ-pretreated MSC (MSC–IFN-γ) on anti-CD40, anti-IgM, and IL-2 stimulated B cells using flow cytometry. After 7 days of co-culturing with MSC or MSC–IFN-γ, viable naïve and memory B cells were distinguished based on intensity of CD27 as shown in Figure 1A.

Co-culture of B cells with MSC significantly increased the proliferation of the total B cell population (Figures 1B,C). MSC that were pretreated with IFN-γ did not increase the proliferation of B cells but, by contrast, inhibited B cell proliferation from 65 to 40%. Naïve and memory B cell subsets (CD27^−^ and CD27^+^, respectively) showed similar increases in proliferation when co-cultured with MSC and inhibition of proliferation when co-cultured with MSC–IFN-γ.

These results show that MSC need to be pre-activated with IFN-γ to bring about their anti-proliferative effect on B cells.

### IFN-γ-Pretreated MSC Inhibit IgG Production by B Cells

**Figure 2:**
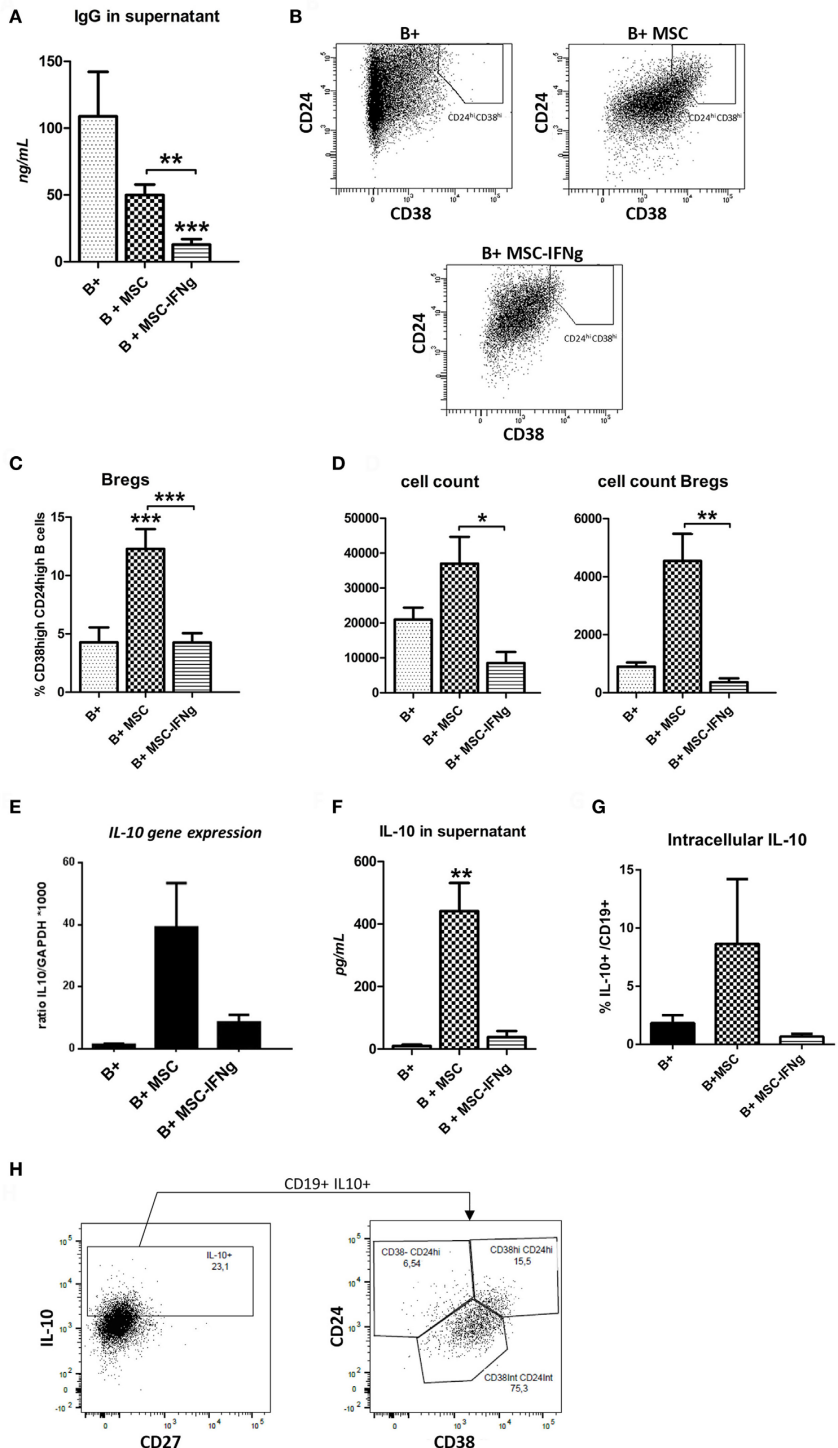
IFN-γ-pretreated mesenchymal stem or stromal cells (MSC) prevent immunoglobulin-G (IgG) production by B cells and regulatory B cell (Breg) formation. **(A)** Levels of IgG were measured in the supernatant of anti-CD40, anti-IgM and IL-2 stimulated B cells co-cultured with or without MSC or MSC– IFN-γ at a 5:1 (B cell:MSC) ratio for 7 days. **(B)** Representative FACS plots of the gating strategy of Bregs, identified as CD38^hi^CD24^hi^ B cells, with or without MSC or MSC–IFN-γ for 7 days. **(C)** Percentage of Bregs of total B cells after culturing with or without MSC or MSC–IFN-γ for 7 days. **(D)** The absolute number of total B cells was counted after harvesting the cells from the co-cultures (left graph). The absolute number of Bregs in the culture was calculated using the percentage of Bregs measured with flow cytometry and the absolute number of total B cells (right graph). Both absolute counts refer to initial 100,000 B cells in culture. **(E)** Gene expression of IL-10 depicted as a ratio to GAPDH. **(F)** Levels of IL-10 were measured in the supernatant of anti-CD40, anti-IgM, and IL-2 stimulated B cells co-cultured with or without MSC or MSC– IFN-γ. **(G)** IL-10 + B cells frequencies measured by analyzing intracellular cytokine by flow cytometry. B cells co-cultured with MSc showed the higher frequencies. **(H)** IL-10 intracellular staining of B cells co-cultured with MSC (B + MSC group). IL-10 positive B cells are plotted to show the percentage of transitional (CD24^hi^ CD38^hi^), CD24^int^ CD38^int^, and CD24^hi^ CD38^−^ subsets. All bars indicate mean ± SEM of three experiments with three different MSC cultures and three different B cell donors.

Mesenchymal stem or stromal cells reduced IgG production by activated B cells (Figure 2A). Pretreatment of MSC with IFN-γ significantly enhanced the inhibitory effect of MSC on IgG production. In accordance with the reduced proliferation, an even stronger reduction of IgG levels was measured in the supernatant of B cells co-cultured with MSC–IFN-γ.

### IFN-γ Conditioned MSC Are Poor Breg Inducers

To investigate whether MSC-induced B cells with a regulatory phenotype, frequencies of CD19^+^ CD38^high^ CD24^high^ transitional B cells (Bregs), and IL-10 production were measured. After 7 days of co-culturing MSC and MSC–IFN-γ with T cell-like stimulated B cells, the percentage of Bregs was measured using flow cytometry as shown in Figure 2B. MSC significantly induced an increase of this subset. By contrast, IFN-γ-pretreated MSC were not able to induce an increase in Bregs (Figures 2B,C). In accordance with this, the absolute number of Bregs was significantly increased when MSC were co-cultured with B cells (Figure 2D).

To analyze whether the induced cells had regulatory potential, the anti-inflammatory cytokine IL-10 gene expression was analyzed. MSC induced a higher trend in IL-10 gene expression, while MSC–IFN-γ did so only to a very low extent (Figure 2E). In accordance with this, IL-10 protein levels were significantly increased in the B cell and MSC co-cultures supernatants, whereas no increase in IL-10 levels was found in the supernatant of B cell and MSC–IFN-γ (Figure 2F). The proportion of IL-10-producing B cells was also analyzed in the different conditions by intracellular staining and accordingly we identified a higher percentage in the co-culture with MSC (Figure 2G).

To further analyze the phenotype of the IL-10-producing B cells induced by MSC we performed intracellular IL-10 staining. The transitional CD38^high^ CD24^high^ subset showed the highest percentage of IL-10^+^ cells, although also within the naïve CD38^int^ CD24^int^ B cell subset significant numbers of IL-10-producing cells were found (data not shown). In absolute numbers, the largest proportion of IL-10-producing B cells was observed in the CD38^int^ CD24^int^ subset, which corresponds to the mature naïve subset. We observed that all IL-10^+^ B cells produced in the presence of MSC were CD27^−^ (Figure 2H).

### The Reduction of B Cell Proliferation by IFN-γ-Pretreated MSC Requires Close Proximity

**Figure 3:**
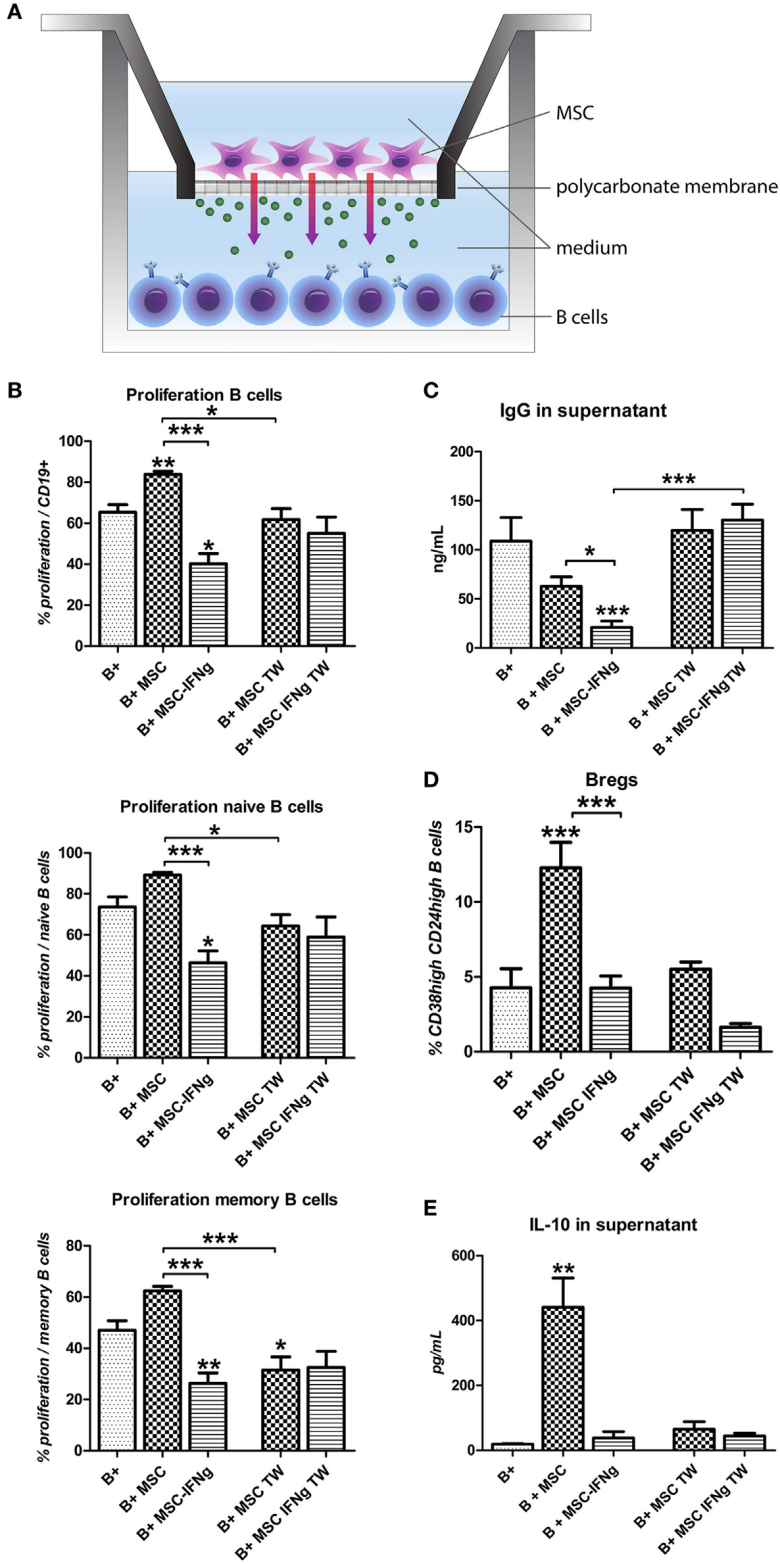
The reduction of B cell proliferation by IFN-γ-pretreated mesenchymal stem or stromal cells (MSC) requires close proximity. **(A)** B cells were stimulated with anti-CD40, anti-IgM, and IL-2 and cultured in direct contact with MSC or MSC– IFN-γ or in transwell (TW) system to prevent direct cell contact of the B cells and MSC. **(B)** Proliferation of B cells was assessed through measurement of 5,6-carboxy-succinimidyl-fluoresceine-ester label dilution. **(C)** Levels of immunoglobulin-G measured in the supernatant with an ELISA assay. **(D)** Percentage of CD24^hi^CD38^hi^ regulatory B cells within CD19 + cell gate measured by flow cytometry. **(E)** Levels of IL-10 measured in the supernatant with an ELISA assay. All bars indicate mean ± SEM of three experiments with three different MSC cultures and three different B cell donors.

To test whether soluble factors or cell contact-dependent mechanisms are involved in the effects of MSC and MSC–IFN-γ on B cell proliferation and Breg induction, activated B cells were co-cultured with MSC and MSC–IFN-γ in a TW system to prevent cell–cell contact as shown in Figure 3A. By preventing direct cell–cell contact, the stimulatory effect of MSC on B cell proliferation was abolished (Figure 3B). Moreover, a small decrease in memory B cell proliferation was measured when B cells were co-cultured with MSC in a TW setting. Interestingly, prevention of direct cell–cell contact also abolished the anti-proliferative capacity of MSC–IFN-γ both in the total B cell population and in the naïve and memory B cell populations (Figure 3B). In accordance with the lack of proliferation inhibition in co-cultures of B cells with MSC–IFN-γ in a TW system, levels of IgG were not affected by MSC and MSC–IFN-γ (Figure 3C). No Bregs were induced when B cells were co-cultured with MSC and MSC–IFN-γ in TW system (Figure 3D), and, in correspondence, no increase in IL-10 levels was found (Figure 3E). These results indicate that the inhibition of B cell proliferation, inhibition of IgG production, and induction of IL-10 production by MSC is dependent on cell contact or at least close proximity of MSC and B cells.

### The Reduction of B Cell Proliferation Requires Metabolically Active MSC–IFN-γ

**Figure 4:**
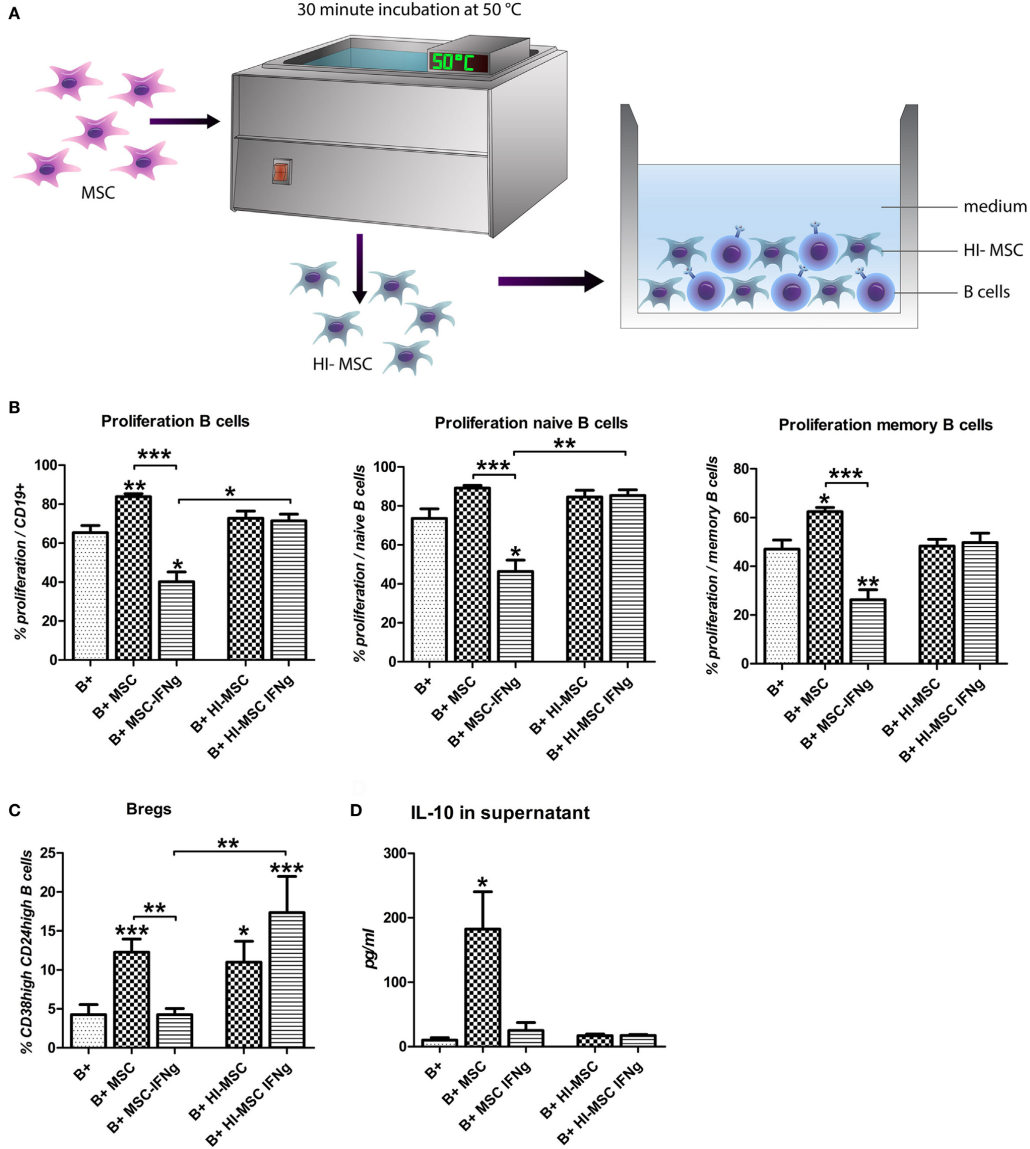
The reduction of B cell proliferation by IFN-γ-pretreated mesenchymal stem or stromal cells (MSC) requires viable cells. **(A)** MSC were incubated for 30 min at 50°C to heat inactivate the cells (HI-MSC). B cells were stimulated with anti-CD40, anti-IgM, and IL-2 and cultured for 7 days with HI-MSC or HI-MSC–IFN-γ. **(B)** Proliferation of B cells was assessed through measurement of 5,6-carboxy-succinimidyl-fluoresceine-ester label dilution. **(C)** Percentage of induced CD24^hi^CD38^hi^ regulatory B cells within CD19 + cell gate measured by flow cytometry. **(D)** The levels of IL-10 were measured in the supernatant of B cells cultured in the presence of viable MSC or MSC– IFN-γ or HI-MSC or HI-MSC–IFN-γ. All bars indicate mean ± SEM of three experiments with three different MSC cultures and three different B cell donors.

To examine whether the inhibition of B cell proliferation by MSC–IFN-γ requires merely interaction via membrane proteins or requires metabolically active MSC–IFN-γ, activated B cells were co-cultured with heat-inactivated MSC (HI-MSC) (Figure 4A). HI-MSC are immunophenotypically intact but release no soluble factors, as previously described (24). Culturing B cells with HI-MSC abolishes the stimulatory effect of MSC on B cell proliferation (Figure 4B) and furthermore the proliferation of B cells was not significantly inhibited by HI-MSC–IFN-γ (Figure 4B). HI-MSC and HI-MSC–IFN-γ induced an increase in Bregs but this increase was not linked to an increase in IL-10 production (Figures 4C,D). These data indicate that the inhibition of B cell proliferation is dependent on metabolic activity of MSC–IFN-γ. Furthermore, the induction of Bregs cannot be recuperated by inactivating MSC–IFN-γ but requires metabolically active MSC. Activating MSC with IFN-γ appears to overrule the Breg inducing capacity of MSC.

### Inhibition of B Cell Proliferation by IFN-γ Stimulated MSC Is Largely Dependent on TRP Catabolism by IDO

**Figure 5:**
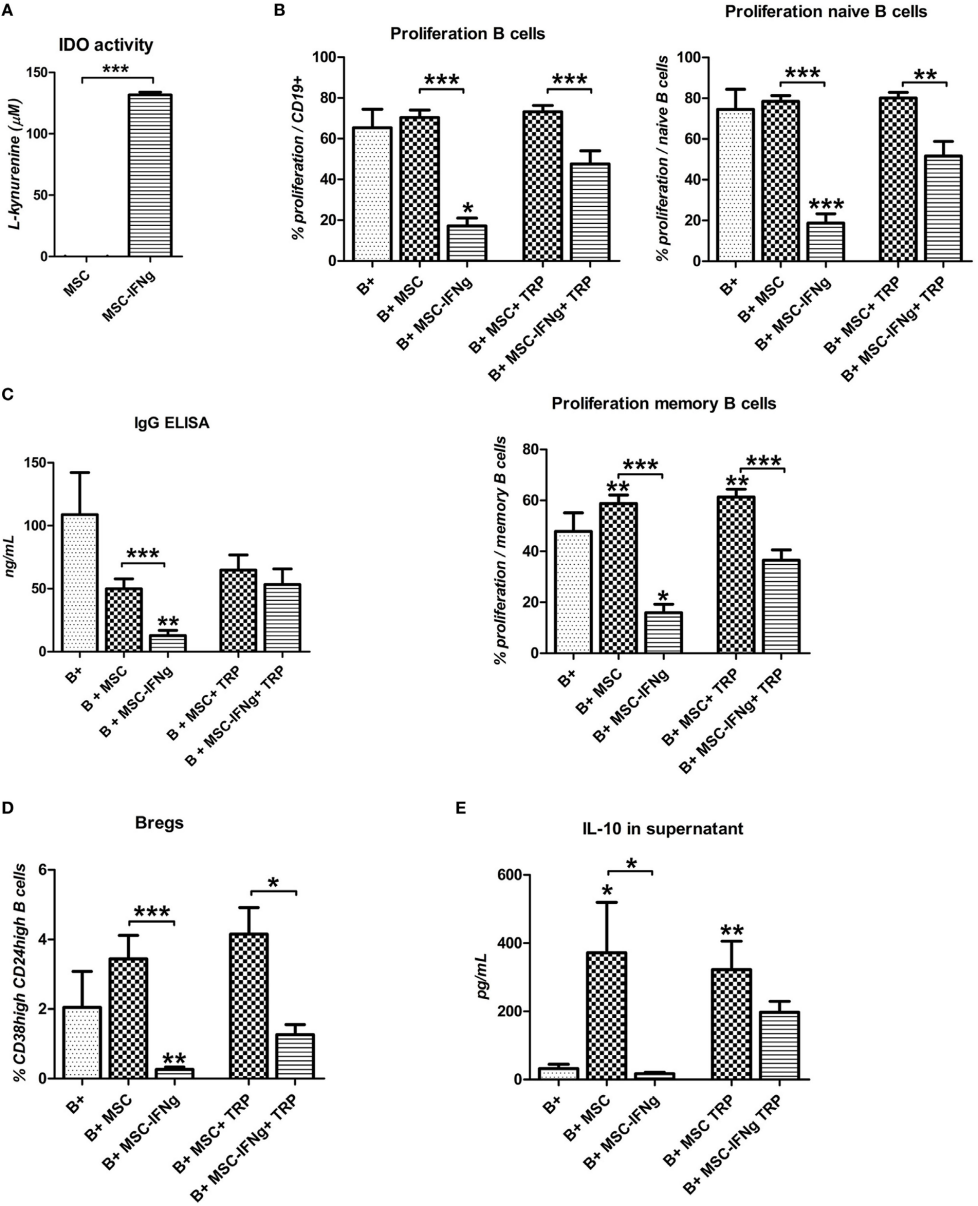
Inhibition of B cell proliferation by IFN-γ stimulated mesenchymal stem or stromal cells (MSC) is largely dependent on tryptophan (TRP) catabolism by indoleamine 2,3-dioxygenase (IDO). **(A)** IDO activity was measured by accumulation of l-kynurenine in MSC supernatant after 4 days culture with or without 50 ng/mL IFN-γ. **(B)** anti-CD40, anti-IgM, and IL-2 stimulated B cells were co-cultured with MSC or MSC–IFN-γ for 7 days in the absence or presence of 200 µM tryptophan (TRP). Proliferation of CFSE labeled B cells is depicted as mean ± SEM of 3 experiments with different MSC cultures. IgG **(C)** and IL-10 **(E)** levels were measured in the supernatant of the cultures using ELISA. **(D)** Percentage of induced regulatory B cells within CD19 + cells with or without added MSC or IFN-γ in the presence or absence of 200 µM TRP for 7 days. All bars indicate mean ± SEM of three experiments with three different MSC cultures and three different B cell donors.

We hypothesized that the inhibition of B cell proliferation by MSC is mediated by IFN-γ triggered IDO induction, leading to degradation and depletion of TRP. When MSC were cultured for 4 days with IFN-γ high levels of l-kynurenine, the breakdown product of TRP, were detected (Figure 5A). When 200 µM TRP was added to B cell and MSC–IFN-γ co-cultures to counteract the effect of IDO activity, B cell proliferation increased from 17 to 48% in the total B cell population, from 19 to 52% in the case of naïve B cell proliferation, and from 16 to 36% in the case of memory B cell proliferation (Figure 5B). TRP supplementation, furthermore, reversed the effect of MSC–IFN-γ on IgG production by B cells (Figure 5C).

### TRP Supplementation Rescues Breg Induction by IFN-γ Stimulated MSC

We showed that IFN-γ-pretreated MSC were not able to induce an increase in Bregs. TRP supplementation to MSC–IFN-γ and B cell co-cultures showed a trend toward increased frequencies of CD38^high^ CD24^high^ Bregs (Figure 5D). In accordance with this, the levels of IL-10 in the supernatant of the MSC–IFN-γ cultures were significantly increased when B cell proliferation was rescued with TRP supplementation (Figure 5E). Stimulation of MSC and MSC–IFN-γ with T cell-like stimulation and TRP did not induce IL-10 secretion by MSC, eliminating the possibility that the IL-10 in the stimulated cultures is secreted by MSC–IFN-γ (*data not shown*). These data indicate that the incapability of MSC–IFN-γ to induce Bregs is caused by TRP depletion mediated by IFN-γ triggered IDO activity in MSC.

## Discussion

**Figure 6:**
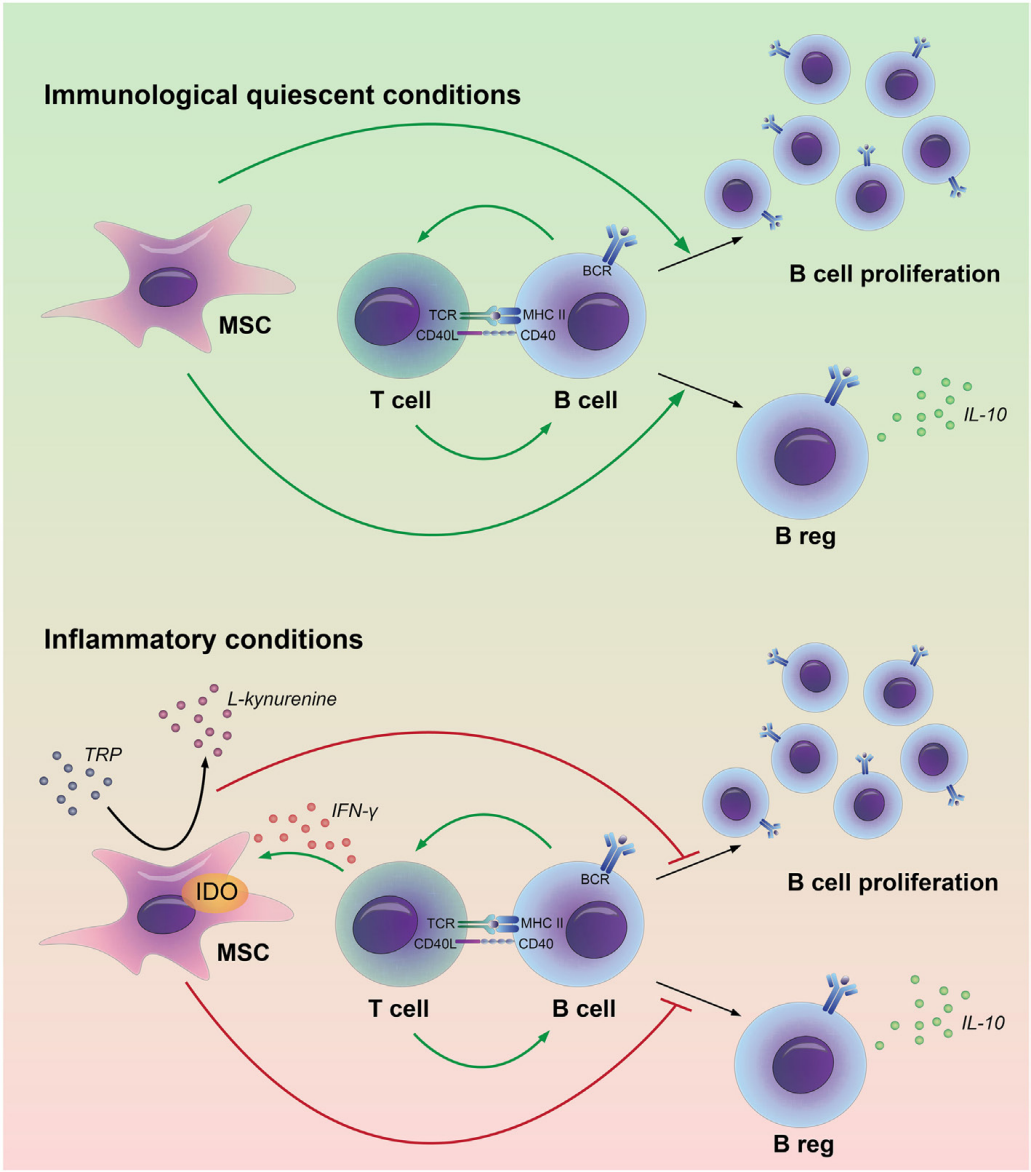
Model for the interactions between adipose tissue-derived mesenchymal stem or stromal cells (MSC) and B cells in immunological quiescent and in inflammatory conditions. MSC have a stimulatory effect on B cell proliferation and regulatory B-cell formation in an immunological quiescent environment. Under inflammatory conditions, MSC break down tryptophan (TRP) through indoleamine 2,3-dioxygenase (IDO). The depletion of TRP leads to an inhibition of B cell proliferation and prevents regulatory B-cell formation.

The immunomodulatory properties of MSC are under strict control of pro-inflammatory factors, such as IFN-γ (13). In this study, we show that inflammatory signals alter the effect of MSC on B cells. In the absence of immune activation, MSC promote the survival of B cells and induce the formation of Bregs, whereas they have little effect on B cell proliferation and IgG production (11). However, after pretreatment with IFN-γ, MSC inhibit B cell proliferation, reduce IgG production, but they also lose the capacity to induce Bregs (Figure 6). During immune responses, immune cells involved in graft rejection such as T cells, monocytes, or macrophages can provide IFN-γ to MSC (26, 27). We previously showed that in the absence of T cells, MSC fail to inhibit activated B cell proliferation (11). Our results indicate that IFN-γ production by T cells is required to activate MSC to dampen the proliferative response of B cells. The decreased levels of IgG and Bregs found when B cells were co-cultured with IFN-γ-stimulated MSC are likely a consequence of the inhibited proliferation of B cells. These data indicate that the effects of MSC on B cells may be very different in situations where no T cells are around, such as, for instance, in patients in which T cells have been depleted with anti-thymocyte globulin after solid organ transplant rejection (28).

The interaction between MSC and B cells has been investigated in a number of studies, although study outcomes have been contrasting with respect to effects of MSC on B cell proliferation and antibody production (29). In this study, we clarified that the effect of MSC on B cells depends on local immunological conditions. Under immunological quiescent conditions, MSC are supportive for B cells; they promote B cell survival and Breg formation. Bregs will subsequently contribute to maintenance of immunological homeostasis. Under inflammatory conditions, in our study mimicked by the addition of IFN-γ, MSC suppress the activity of B cells; they inhibit B cell proliferation and reduce antibody production. At the same time, they inhibit Bregs induction. This may seem counterintuitive, but may reflect a state in which all B cell activity is shut down by MSC. Our results imply that *in vivo*, resident MSC are supportive for B cells and induce tolerogenic B cells under immunological quiescent conditions, whereas under inflammatory conditions MSC suppress humoral responses. For the generation of therapeutic MSC our results suggest that custom-made MSC can be generated with either B cell suppressive properties or with B cell homeostasis supportive properties. Distinct mechanisms have been described to be responsible for immunomodulation by MSC. Both soluble factors and contact-dependent ligand–receptor interaction have been proposed to participate to the MSC-mediated immunomodulation (30). We show that MSC effects on B cells do not solely depend on soluble factors as no Bregs or IL-10 production were induced when MSC were cultured in a TW culture system. Moreover, the presence of dead but phenotypically intact MSC (24) was not enough to induce IL-10-producing B cells, implying that modulation of B cells by MSC is mediated by an active metabolic process and needs close proximity of MSC and B cells.

Indoleamine 2,3-dioxygenase-mediated TRP catabolism has been described as an important mechanism of activated MSC to modulate T cell proliferation (17). We demonstrated that the inhibition of B cell proliferation by MSC also largely depends on the TRP depleting activity of IDO activity and can be recovered by supplementing TRP *in vitro*. We show that the ability of MSC to induce IL-10-producing B cells was lost when MSC were pretreated with IFN-γ but could be recovered when TRP was supplemented to the culture. Thus, in the tested experimental conditions, MSC–IFN-γ act in a similar way to non-activated MSC upon TRP supplementation, indicating that IFN-γ-induced IDO activity plays a major role in the effect of IFN-γ-activated MSC on B cells.

In this study, we named the transitional B cell subset characterized by CD19^+^ CD24^hi^ CD38^hi^ as Bregs, since this is one of the most commonly used phenotypes for this subset of B cells in humans and we have previously proven it is consistently upregulated in the presence of MSC. We further characterized this subset by quantification of IL-10 production as IL-10 is the most widely used to define Bregs function. The definition of the Breg population is an important discussion point in our manuscript and in current literature. There is no unique signature that identifies the Breg subset and probably there are many different Breg subsets with different phenotypes. In our setting, we have previously observed that MSC increase the proportion of naïve (CD19^+^ CD27^−^) and transitional (CD19^+^ CD24^high^ CD38^high^) B cells, which was correlated to an increase of IL-10 gene expression and protein production (11). However, the intracellular IL-10 staining in this study reveals that there is no complete match between the transitional B cells immunophenotype and IL-10-producing cells, so further marker discovery is needed to unravel a more suitable signature or a master transcription factor that would allow to properly label Bregs. While such key markers are not discovered, we used both the transitional B cell immunophenotype and the amount of IL-10 released in the culture medium to semi-quantify the Breg population in this study.

Better understanding of the interaction between MSC and B cells under different immunological conditions is important for designing therapeutic approaches targeting B cells using MSC. Conventional MSC therapy can potentially be used to induce Breg formation and thereby promote tolerance such as after organ transplantation. Peng et al. show that MSC therapy in chronic graft versus host disease patients led to increased number of IL-10-producing CD5^+^ Bregs and increased IL-10 production by these cells (31). On the other hand, IFN-γ-activated MSC as therapy could be beneficial in B cell-mediated diseases where suppression of B cell proliferation and IgG production is desired.

To summarize, we show that immunological conditions can dictate the effect of MSC on B cell function. MSC induce B cells with a regulatory phenotype but are not capable to dampen B cell proliferation. Under T cell-mediated inflammatory conditions, MSC strongly inhibit B cell proliferation and, as a consequence, IgG production although they do not induce formation of Bregs. This shows for the first time that MSC adapt their effect on B cells to the inflammatory climate. *In vivo* this means that resident MSC are supportive for B cells and induce tolerogenic B cells under immunological quiescent conditions, whereas under inflammatory conditions MSC suppress humoral responses. For therapeutic MSC, this means that we can generate MSC with either B cell suppressive properties, or MSC that support B cell homeostasis. With this knowledge specific MSC therapy can be designed for different immune disorders or transplantation.

## Author Contributions

FL: collection of data, data analysis and interpretation, and manuscript writing. LC-P: collection of data, data analysis and interpretation, and final approval of manuscript. SK: collection of data, data analysis and interpretation, and final approval of manuscript. SW: collection of data, data analysis and interpretation, and final approval of manuscript. FB: final approval of manuscript. MB and CB: data analysis and interpretation, and final approval of manuscript. MH: conception and design, data analysis and interpretation, and manuscript writing. MF: conception and design, collection of data, data analysis and interpretation, and manuscript writing.

## Conflict of Interest Statement

The authors declare that the research was conducted in the absence of any commercial or financial relationships that could be construed as a potential conflict of interest.

## Supplementary Material

Figure S1 Characterization of MSC before and after culturing with IFN*-*γ. MSC were cultured for 4 days with IFN-γ (50 ng/ml). **(A)** Bright-field image of cultured MSC shows characteristic MSC morphology before and after culturing with IFN-γ. **(B)** The presence of CD13, CD73, CD90, CD105, HLA-ABC, HLA-DR, and PD-L1 and absence of CD45 and CD31 cell surface markers measured on MSC before *(solid line)* and after culturing with IFN-γ *(dotted line)*. Gray, solid histograms represent unstained MSC.
