# Supplementary figures and images for: Inflammatory Conditions Dictate the Effect of Mesenchymal Stem or Stromal Cells on B Cell Function
Source: Front Immunol. 2017 Aug 28;8:1042. doi: 10.3389/fimmu.2017.01042 (PMC5581385; doi:10.3389/fimmu.2017.01042)

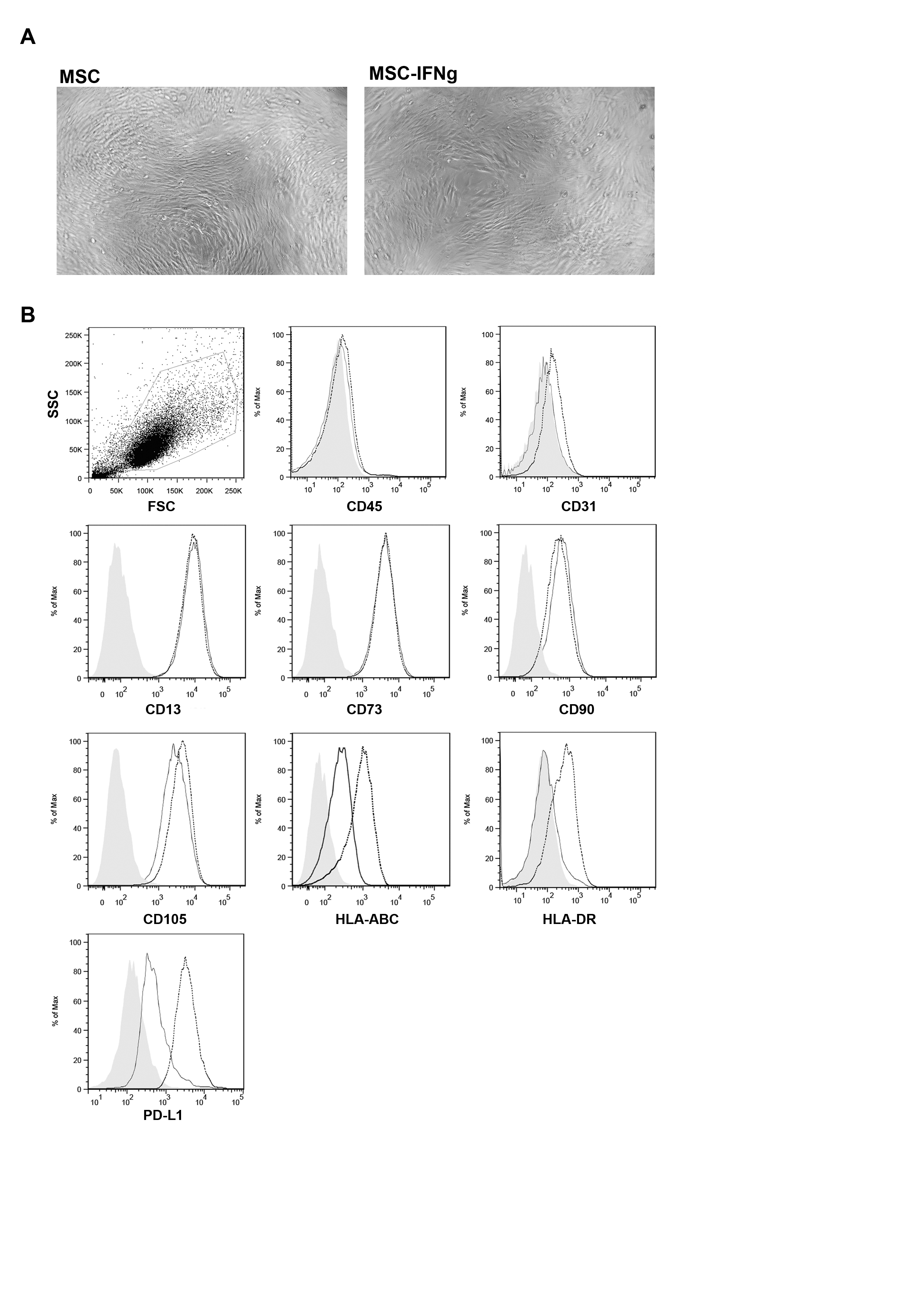

Supplement: Figure S1 — Characterization of MSC before and after culturing with IFN-γ. MSC were cultured for 4 days with IFN-γ (50 ng/ml). (A) Bright-field image of cultured MSC shows characteristic MSC morphology before and after culturing with IFN-γ. (B) The presence of CD13, CD73, CD90, CD105, HLA-ABC, HLA-DR, and PD-L1 and absence of CD45 and CD31 cell surface markers measured on MSC before (solid line) and after culturing with IFN-γ (dotted line). Gray, solid histograms represent unstained MSC. [file Image_1.tif]
